# Exploratory classification of clinical phenotypes in Japanese patients with antineutrophil cytoplasmic antibody-associated vasculitis using cluster analysis

**DOI:** 10.1038/s41598-021-84627-6

**Published:** 2021-03-04

**Authors:** Haruki Watanabe, Ken-ei Sada, Masayoshi Harigai, Koichi Amano, Hiroaki Dobashi, Yoshinari Takasaki, Shouichi Fujimoto, Tatsuya Atsumi, Kunihiro Yamagata, Sakae Homma, Yoshihiro Arimura, Hirofumi Makino, Research Committee of Intractable Vasculitis Syndrome (JPVAS), Research Committee of Intractable Renal Disease of the Ministry of Health, Labour, and Welfare of Japan

**Affiliations:** 1 Department of Nephrology, Rheumatology, Endocrinology and Metabolism, Okayama University Graduate School of Medicine, Dentistry and Pharmaceutical Sciences, 2-5-1 Shikata-cho, Kita-ku, Okayama, 700-8558 Japan; 2 Department of Rheumatology, Tokyo Women’s Medical University School of Medicine, Tokyo, Japan; 3 Department of Rheumatology and Clinical Immunology, Saitama Medical Center, Saitama Medical University, Kawagoe, Japan; 4 Division of Hematology, Rheumatology and Respiratory Medicine, Department of Internal Medicine, Faculty of Medicine, Kagawa University, Kita-gun, Miki-cho, Japan; 5 Department of Internal Medicine and Rheumatology, Juntendo University School of Medicine, Tokyo, Japan; 6 Department of Hemovascular Medicine and Artificial Organs, Faculty of Medicine, University of Miyazaki, Miyazaki, Japan; 7 Department of Rheumatology, Endocrinology and Nephrology, Faculty of Medicine and Graduate School of Medicine, Hokkaido University, Sapporo, Japan; 8 Department of Nephrology, Faculty of Medicine, University of Tsukuba, Ibaraki, Japan; 9 Department of Advanced and Integrated Interstitial Lung Diseases Research, School of Medicine, Toho University, Tokyo, Japan; 10 Department of Nephrology and Rheumatology, Kyorin University School of Medicine, Tokyo, Japan; 11 Kichijoji Asahi Hospital, Tokyo, Japan; 12 Okayama University, Okayama, Japan

**Keywords:** Rheumatology, Rheumatic diseases

## Abstract

A novel patient cluster in antineutrophil cytoplasmic antibody (ANCA)-associated vasculitis (AAV) may be identified in Japan. We performed multiple correspondence and cluster analysis regarding 427 clinically diagnosed AAV patients excluding eosinophilic granulomatosis with polyangiitis. Model 1 included the ANCA phenotype, items of the Birmingham Vasculitis Activity Score, and interstitial lung disease; model 2 included serum creatinine (s-Cr) and C-reactive protein (CRP) levels with model 1 components. In seven clusters determined in model 1, the ANCA-negative (n = 8) and proteinase 3-ANCA-positive (n = 41) groups emerged as two distinct clusters. The other five myeloperoxidase-ANCA-positive clusters were characterized by ear, nose, and throat (ENT) (n = 47); cutaneous (n = 36); renal (n = 256), non-renal (n = 33); and both ENT and cutaneous symptoms (n = 6). Four clusters in model 2 were characterized by myeloperoxidase-ANCA negativity (n = 42), without s-Cr elevation (< 1.3 mg/dL) (n = 157), s-Cr elevation (≥ 1.3 mg/dL) with high CRP (> 10 mg/dL) (n = 71), or s-Cr elevation (≥ 1.3 mg/dL) without high CRP (≤ 10 mg/dL) (n = 157). Overall, renal, and relapse-free survival rates were significantly different across the four clusters in model 2. ENT, cutaneous, and renal symptoms may be useful in characterization of Japanese AAV patients with myeloperoxidase-ANCA. The combination of s-Cr and CRP levels may be predictive of prognosis.

## Introduction

Antineutrophil cytoplasmic antibody (ANCA)-associated vasculitis (AAV) is a multisystem autoimmune disease characterized by ANCA production and small- and medium-sized blood vessel inflammation^1^. Eosinophilic granulomatosis with polyangiitis (EGPA), Granulomatosis with polyangiitis (GPA), and microscopic polyangiitis (MPA), are the major categories of AAV, and proteinase 3 (PR3) and myeloperoxidase (MPO) are two major antigens of ANCA^2^. PR3-ANCA is generally regarded as a marker for granulomatosis with polyangiitis (GPA)^2^, while MPO-ANCA is for microscopic polyangiitis (MPA)/renal-limited vasculitis^3^. Though various classification and diagnostic criteria combining clinical symptoms and ANCA phenotypes have been used in the clinical study and practice, unclassifiable patients are still remain^3^. Moreover, a recent genome-wide association study has revealed that the ANCA phenotype better classified patients with GPA and MPA than clinical classification, suggesting that better classification should be required^4^.

Cluster analysis is a statistical method of exploratory data mining for grouping objects into homogenous groups by their similarity. A previous report using cluster analysis for AAV suggested that ANCA phenotype and renal involvement could better predict prognosis than clinical classification such as granulomatosis with polyangiitis (GPA) and MPA^5^. We previously have reported predominance of MPA and MPO-ANCA positivity as common characteristics in Japan and other East Asian countries^6^, which is in marked contrast to the results of studies previously reported from Western countries^7,8^. Based on the above mentioned genetic study, there should be marked differences between the genetic backgrounds of AAV patients in Japan and Western countries^4^. Thus, another relevant cluster may be determined in Japan where MPO-ANCA and MPA are dominant among AAV patients.

Though prognostic factors in AAV have not yet fully explored, the Remission Induction Therapy in Japanese Patients with AAV (RemIT-JAV) study revealed that the European Vasculitis Study Group (EUVAS) criteria for disease severity was useful for predicting the prognosis of Japanese patients with AAV^6^, while the Remission Induction Therapy in Japanese Patients with AAV and Rapidly Progressive Glomerulonephritis (RemIT-JAV-RPGN) study added the superior suitability of the Japanese RPGN clinical grading system compared to the EUVAS criteria for disease severity^8^. The Japanese RPGN clinical grading system consists of four components: age, serum creatinine (s-Cr) levels, lung complication, and C-reactive protein (CRP) levels. CRP levels have been reported to be useful for predicting mortality and for distinguishing between active AAV and remission^9,10^. However, no study has elucidated the characteristics of AAV that are represented by CRP levels.

The objectives of the present study were (1) to explore novel clinical groups of MPA, GPA, and unclassifiable patients using cluster analysis in terms of clinical phenotype or severity assessment; (2) to evaluate the associations between the determined clusters and clinical outcomes; and (3) to elucidate the characteristics of AAV associated with CRP levels.

## Methods

### Database

This study used data from the RemIT-JAV study and RemIT-JAV-RPGN study. Twenty-two tertiary care institutions (university hospitals and referring hospitals) participated in RemIT-JAV, and 53 participated in RemIT-JAV-RPGN. The patients with newly diagnosed AAV were prospectively enrolled from April 2009 to December 2010 into RemIT-JAV and from April 2011 to March 2014 into RemIT-JAV-RPGN. There is not an overlap of patients between the two studies. The criteria for enrolment in RemIT-JAV and RemIT-JAV-RPGN were as follows: (1) clinical diagnosis of AAV by the site investigators, (2) fulfillment of the criteria for primary systemic vasculitis, as proposed by the European Medicines Agency (EMEA) algorithm^11^, and (3) initiation of immunosuppressive treatment based on the discretion of the site investigators. The exclusion criteria in RemIT-JAV and RemIT-JAV-RPGN were (1) an age less than 20 years, (2) serological evidence of hepatitis B or C virus infection, and (3) a history of malignancy. The classification of AAV was conducted based on EMEA algorithm^11^.

### Clinical variables

Data from the two studies were merged in a single dataset. Forty-two patients with EGPA were excluded because EGPA phenotypes are markedly different from phenotypes presented by other types of AAV patients. We also excluded eight patients whose PR3-ANCA results were unavailable at diagnosis.

In the present study, nine items of the Birmingham Vasculitis Activity Score (BVAS) 2003, ILD, s-Cr levels, serum CRP levels, and the ANCA phenotype (proteinase-3 (PR3)-ANCA or MPO-ANCA) were used as clinical variables. Interstitial lung disease (ILD) was confirmed radiologically. The BVAS 2003 includes following symptoms: general; cutaneous; mucous membrane/eye; ear, nose and throat (ENT); chest; cardiovascular; abdominal; renal; and nervous system symptoms^12^. ILD was selected as a candidate clinical variable because of the high prevalence in our cohorts^3,8^. The patients enrolled in RemIT-JAV and RemIT-JAV-RPGN were evaluated at 3, 6, 12, 18, and 24 months after diagnosis and at the time of relapse. We collected the following outcome measures: remission rate, overall survival rate, end-stage renal disease (ESRD)-free survival rate, and relapse rate. Remission was defined as BVAS = 0 (new or worse) on two consecutive occasions that occurred at least one month apart^13^. ESRD was defined as dependence on dialysis or an irreversible increase in s-Cr level of > 5.6 mg/dL (500 μmol/L)^14^. Relapse was defined as the recurrence or new onset of clinical signs and symptoms attributable to active vasculitis^15^.

### Statistical analysis

Cluster analysis was performed based on two models. Model 1 included the nine clinical symptoms considered in the BVAS, PR3-ANCA and MPO-ANCA, and ILD for assessment of the clinical phenotype. Model 2 included laboratory data for two additional characteristics (s-Cr and CRP levels) for the assessment of disease severity^8–10^. The s-Cr levels were categorized on the basis of the thresholds of the EUVAS criteria for disease severity (1.3 mg/dL (120 μmol/L) and 5.6 mg/dL (500 μmol/L))^15^, and the CRP levels were categorized on the basis of the Japanese RPGN clinical grading system (2.6 mg/dL and 10.0 mg/dL), which could stratify the prognosis in patients with AAV and/or RPGN^8,16^. There are no missing data regarding these variables among enrolled patients.

At first, multiple correspondence analysis was performed to select candidate variables. Using principal component analysis (PCA), the contribution rate of each variable was calculated according to the distance from principal component 1 and principal component 2. Variables that explained at least 90% of the total contribution rates were included for the cluster analysis. Subsequently, hierarchical clustering based on the Ward method, followed by consolidation (K-means algorithm), was performed using the determined variables. To decide the optimal number of clusters, a dendrogram was plotted in each model. We determined the clusters by the branches and vertical distance based on each dendrogram. A dominant clinical feature (> 75% or 0% of patients in each cluster) was used to name each cluster.

For evaluation of the discrimination ability of the determined characteristics in each cluster, classification tree analysis was conducted subsequently. The predictive accuracies of the algorithms were calculated using the observed numbers of individuals allocated to the predicted classes. The overall survival, ESRD-free survival, cumulative remission, and relapse rates were analyzed using the Kaplan–Meier method and the log-rank test across the determined clusters.

To explore the clinical symptoms associated with the serum CRP levels, multiple linear regression analysis was performed using stepwise backward selection to minimize the Bayesian information criterion. Among all 63 items of the BVAS and ILD, the items observed in > 5% of enrolled patients were used as candidate variables. The prevalence of each BVAS item or ILD is shown in Supplementary Table S1.

All statistical analyses were performed by a biostatistician using the JMP version 10.0.2 statistical package for Windows (SAS Institute Inc., Cary, NC, USA) or R 3.2.3 (R Foundation for Statistical Computing, Vienna, Austria). A two-tailed *P* < 0.05 was considered statistically significant. When comparing seven or four clusters, the statistical significance was determined by *P* < 0.05/7 or *P* < 0.05/4 by the Bonferroni correction to adjust for multiple testing.

### Ethics approval and consent to participate

This study was approved by the Ethics Committee of the Okayama University Graduate School of Medicine, Dentistry and Pharmaceutical Sciences (authorization number: No. 1909-016), and conducted according to the Declaration of Helsinki and the Ethical Guidelines for Epidemiological Research in Japan. Written informed consent was obtained from each participant, and the study protocol was approved by the ethics committee of each participating hospital. The RemIT-JAV study and RemIT-JAV-RPGN study were registered with the University Hospital Medical Information Network Clinical Trials Registry (UMIN000001648 and 000005136).

## Results

### Patient characteristics and clinical outcomes

Of 477 patients with AAV enrolled in the two cohort studies, 427 patients were enrolled in the present study (142 patients from the RemIT-JAV and 285 patients from the RemIT-JAV-RPGN study, Supplementary Fig. S1). The enrolled patient characteristics are shown in Supplementary Table S2. Among enrolled 427 patients, fifteen patients showed positive results for both types of ANCA. Remission was achieved in 88% (n = 376) of the enrolled patients, and relapses occurred among 15% (n = 57) of the remitted patients. During the median (IQR) observational periods of 730 (654–730) days, 47 deaths and 46 ESRDs were reported.

### Cluster analysis in model 1

**Figure 1:**
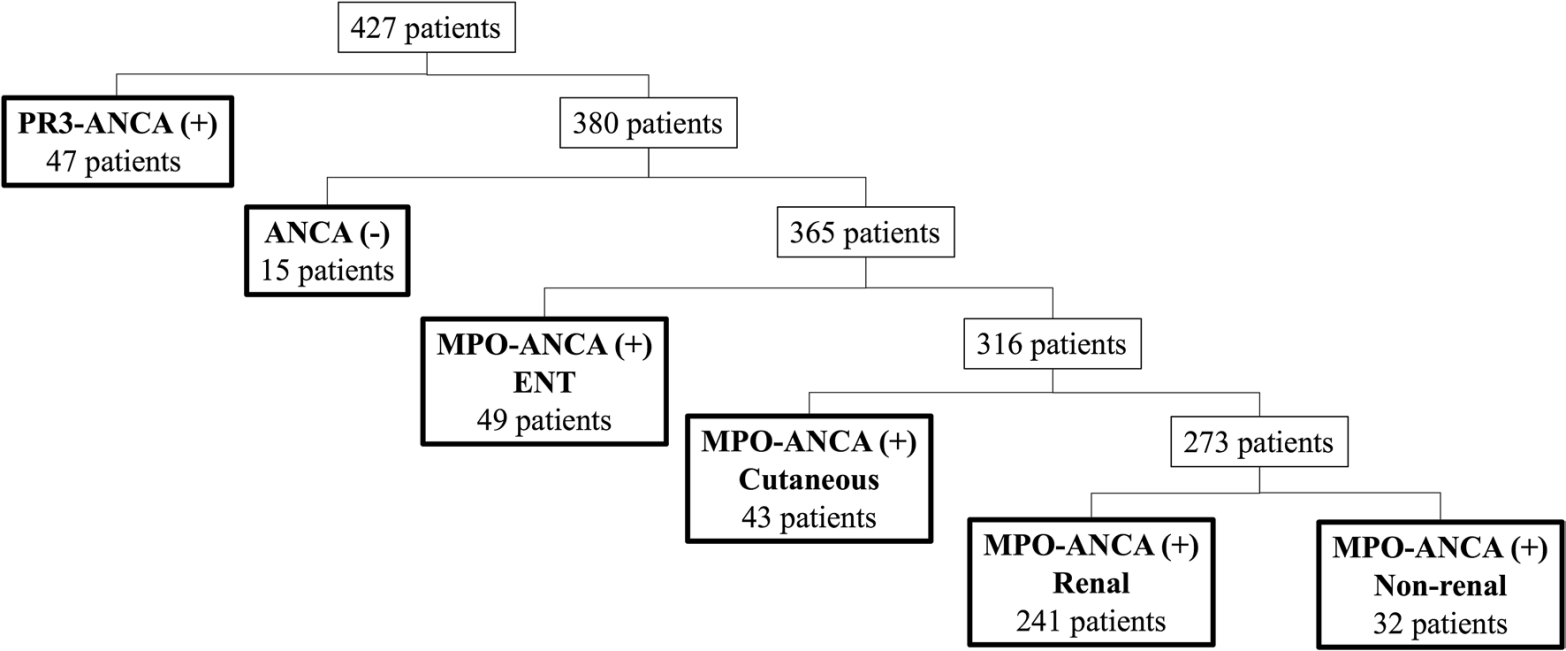
Classification tree analysis of 427 patients with antineutrophil cytoplasmic antibody-associated vasculitis in model 1. The dominant clinical feature (over 80%) is used to name each class. The observed number of individuals allocated to each class is shown in each column. The algorithm was initiated on the basis of ANCA positivity and was subsequently allocated according to organ involvement. Among 47 patients with PR3-ANCA, 34 were assigned to Cluster 1. Among 15 patients with ANCA negativity, 8 were assigned to Cluster 2. Among 49 patients with MPO-ANCA and ENT symptoms, 48 were assigned to Cluster 3 or 7. Among 43 patients with MPO-ANCA and skin symptoms, 34 were assigned to Cluster 4. The rest were assigned to predicted clusters by cluster analysis. The overall concordance rate was 93%. *ENT* ear, nose and throat, *ILD* interstitial lung disease, *MPO-ANCA* myeloperoxidase-antineutrophil cytoplasmic antibody, *PR3-ANCA* proteinase-3-antineutrophil cytoplasmic antibody.

On the basis of the contribution rates of the candidate variables in model 1 by PCA, eight variables were selected with 91% of total contribution rates: MPO-ANCA, PR3-ANCA, ENT symptoms, nervous system symptoms, general symptoms, renal symptoms, cutaneous symptoms, and ILD (Supplementary Table S3). By the dendrogram of model 1, seven clusters were suggested (Supplementary Fig. S2). Patient characteristics were compared across seven clusters, as presented in Table 1. Cluster 1 was characterized by PR3-ANCA positivity (34 of 41, 82.9%) and Cluster 2 by ANCA negativity (8 of 8, 100%). All patients in the other five clusters were MPO-ANCA-positive. Among the five clusters of MPO-ANCA-positive patients, Cluster 3 was characterized by ENT symptoms (47 of 47, 100%), Cluster 4 by cutaneous symptoms (36 of 36, 100%), and Cluster 5 by renal symptoms (256 of 256, 100%). No patient in Cluster 6 had renal symptoms. All patients in Cluster 7 had both ENT and cutaneous symptoms. Classification tree analysis based on specified characteristic in each cluster is presented in Fig. 1. First, 47 patients were classified into the PR3-ANCA-positive group. Then, 15 patients were classified into the ANCA-negative group. Among the MPO-ANCA-positive patients, 49 patients were classified as having ENT symptoms, 43 patients were classified as the cutaneous group, 241 patients as the renal group (all patients were in Cluster 5), and the remaining patients exhibited no renal symptoms (i.e., non-renal group). In 397 (93%) patients, the classification tree analysis showed consistent results with cluster analysis when Cluster 7 (ENT with cutaneous symptoms) was combined with Cluster 3 (ENT symptoms).

**Table 1:** Comparison of patient characteristics across seven clusters based on model 1.

	Cluster 1 (n = 41) PR3-ANCA (+)	Cluster 2 (n = 8) ANCA (−)	Cluster 3 (n = 47) MPO-ANCA (+) ENT	Cluster 4 (n = 36) MPO-ANCA (+) Cutaneous	Cluster 5 (n = 256) MPO-ANCA (+) Renal	Cluster 6 (n = 33) MPO-ANCA (+) Non-renal	Cluster 7 (n = 6) MPO-ANCA (+) ENT with cutaneous
Female/male	22/19	3/5	28/19	22/14	141/115	23/10	4/2
Age (years)	63 (58–74)	64 (60–73)	72 (62–79)	69 (59–76)	73 (65–79)	75 (68–80)	71 (62–75)
GPA (%)	34 (83)	2 (25)	34 (72)	2 (6)	11 (4)	1 (3)	2 (33)
MPA (%)	3 (7)	2 (25)	12 (26)	25 (69)	218 (85)	7 (21)	3 (50)
Unclassifiable (%)	4 (10)	4 (50)	1 (2)	9 (25)	27 (11)	25 (76)	1 (17)
MPO**-**ANCA (%)	2 (5)	0	47 (100)	36 (100)	256 (100)	33 (100)	6 (100)
PR3-ANCA (%)	34 (82.9)	0	4 (8.5)	1 (2.8)	7 (2.7)	0	1 (16.7)
Serum creatinine (mg/dL)	0.79 (0.6–1.2)	0.64 (0.57–0.71)	1.2 (0.83–3.5)	0.96 (0.66–19)	2.1 (0.97–4.0)	0.67 (0.57–0.82)	0.99 (0.75–1.6)
CRP (mg/dL)	3.9 (1.2–11)	4.3 (1.2–11)	11 (5.6–15)	7.4 (2.6–12)	6.5 (1.4–12)	8.1 (4.6–12)	9.3 (0.44–16.3)
ILD (%)	3 (7)	3 (38)	12 (26)	8 (22)	126 (49)	28 (85)	5 (83)
General (%)	26 (63)	8 (100)	37 (79)	29 (81)	155 (61)	25 (76)	4 (67)
Cutaneous (%)	4 (10)	4 (50)	0	36 (100)	8 (3)	1 (3)	6 (100)
Mucous membranes/eyes (%)	15 (37)	1 (13)	14 (30)	2 (6)	16 (6)	2 (6)	0
Ear nose and throat (%)	33 (80)	2 (25)	47 (100)	1 (3)	0	0	6 (100)
Chest (%)	18 (44)	2 (25)	29 (62)	13 (36)	98 (38)	9 (27)	3 (50)
Cardiovascular (%)	3 (7)	0	3 (6)	3 (8)	25 (10)	0	0
Abdominal (%)	1 (2)	0	0	0	3 (1)	0	0
Renal (%)	22 (54)	1 (13)	37 (79)	28 (78)	256 (100)	0	4 (67)
Nervous system (%)	12 (29)	7 (88)	21 (45)	21 (58)	62 (24)	9 (27)	4 (67)

Values expressed as the number of patients or median (interquartile).

*CRP* C-reactive protein, *GPA* granulomatosis with polyangiitis, *ILD* interstitial lung disease, *MPA* microscopic polyangiitis, *MPO-ANCA* myeloperoxidase-antineutrophil cytoplasmic antibody, *PR3-ANCA* proteinase-3-antineutrophil cytoplasmic antibody.

The overall survival rate was not significantly different across the clusters in model 1 (*P* = 0.325, Supplementary Fig. S3A). Cluster 4 (MPO-ANCA-positive patients with cutaneous disease) tended to show worse survival, but not significantly worse survival (*P* = 0.026), compared to Cluster 1 (PR3-ANCA-positive patients, Supplementary Fig. S3A). The ESRD-free survival rate differed significantly across the seven clusters (*P* = 0.039), but no significant differences were found between any two clusters (Supplementary Fig. 3B). Cumulative remission (Supplementary Fig. S4A) and relapse-free survival (Supplementary Fig. 4B) rates were not significantly different across the clusters in model 1 (*P* = 0.239 and 0.250, respectively).

### Cluster analysis in model 2

**Figure 2:**
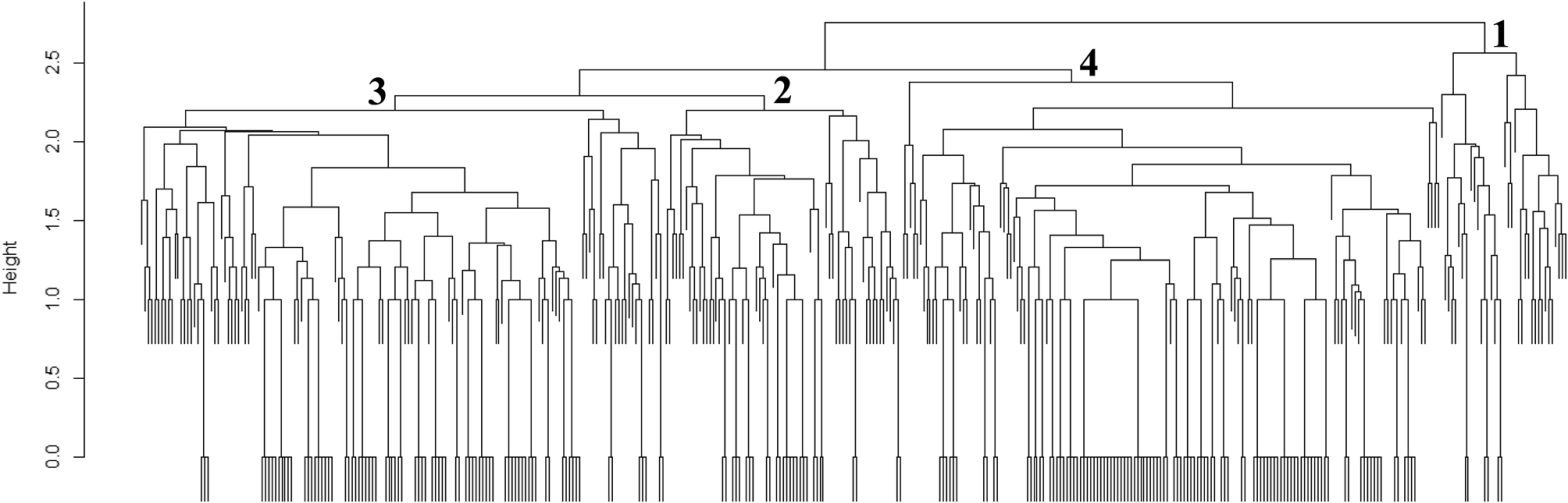
Dendrograms for cluster model 2 for antineutrophil cytoplasmic antibody-associated vasculitis. The dendrogram shows the clustering process of model 2 resulting in four clusters. Cluster 1 was characterized by MPO-ANCA negativity, Cluster 2 by s-Cr elevation with high CRP (CRP > 10 mg/dL and creatinine ≥ 1.3 mg/dL), Cluster 3 by without s-Cr elevation (s-Cr < 1.3 mg/dL), and Cluster 4 by s-Cr elevation (s-Cr ≥ 1.3 mg/dL) without high CRP (≤ 10 mg/dL). *MPO-ANCA* myeloperoxidase-antineutrophil cytoplasmic antibody, *CRP* C-reactive protein, *s-Cr* serum creatinine.

**Figure 3:**
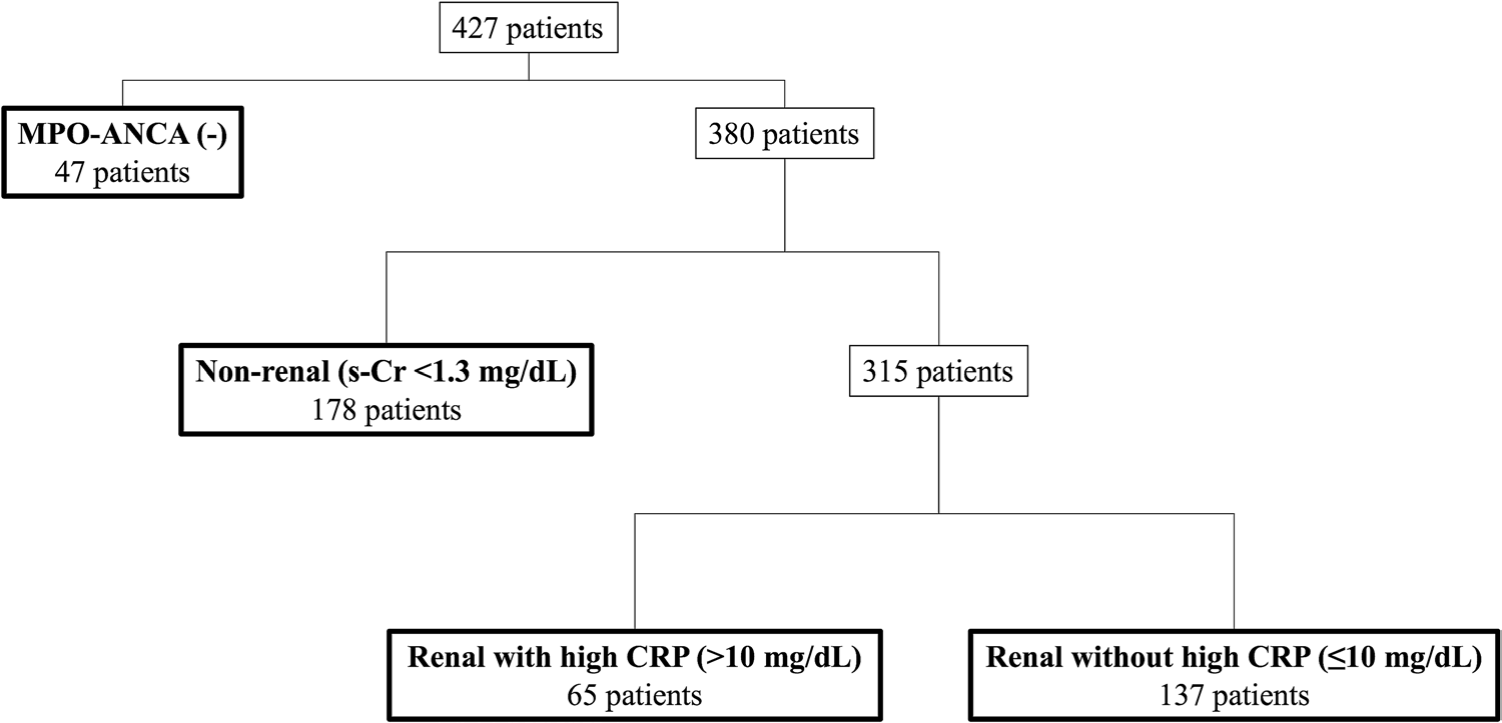
Classification tree analysis of 427 patients with antineutrophil cytoplasmic antibody-associated vasculitis in model 2. The dominant clinical feature (over 75%) is used to name each class. The observed number of individuals allocated to each class is shown in each column. The algorithm was initiated on the basis of MPO-ANCA positivity and was subsequently allocated by s-Cr and CRP levels. Among 47 patients with MPO-ANCA negativity, 39 were assigned to Cluster 1. Among 178 MPO-ANCA-positive patients without s-Cr elevation, 133 were assigned to Cluster 3. Among 65 MPO-ANCA-positive patients with s-Cr elevation and high CRP, 54 were assigned to Cluster 3. Among the remining 137 patients with s-Cr elevation and without high CRP, 117 were assigned to Cluster 4. The overall concordance rate was 80%. *CRP* C-reactive protein, *MPO-ANCA* myeloperoxidase-antineutrophil cytoplasmic antibody, *s-Cr* serum creatinine.

Next, PCA of model 2 (including the CRP and s-Cr levels) was performed, and nine variables were selected with a total of 93% contribution rates: MPO-ANCA, PR3-ANCA, general symptoms, ENT symptoms, CRP, nervous system symptoms, creatinine, mucous membrane/eye symptoms; and renal symptoms (Supplementary Table S4). By the dendrogram of model 2, four clusters were suggested (Fig. 2). Patient characteristics were compared across four clusters, as presented in Table 2. Cluster 1 was characterized by MPO-ANCA negativity (39 of 42, 93%), Cluster 2 by s-Cr elevation (≥ 1.3 mg/dL (115 μmol/L)) with high CRP (> 10 mg/dL) (54 of 71, 76%; renal with high CRP), Cluster 3 by without s-Cr elevation (< 1.3 mg/dL) (10 of 157, 89%; non-renal), and Cluster 4 by s-Cr elevation (≥ 1.3 mg/dL) without high CRP (≤ 10 mg/dL) (117 of 157, 75%; renal without high CRP). Classification tree analysis based on specified characteristics in each cluster is presented in Fig. 3. First, 47 patients were classified as MPO-ANCA-negative. Among the MPO-ANCA-positive patients, 178 patients were classified as a non-renal group, 65 patients as a renal group with high CRP level, and 137 patients as a renal group without high CRP level. Classification tree analysis was consistent with cluster analysis in model 2; 343 (80%) patients were classified in the same cluster.

**Table 2:** Comparison of patient characteristics across four clusters based on model 2.

	Cluster 1 MPO-ANCA (−) (n = 42)	Cluster 2 Renal with high CRP (n = 71)	Cluster 3 Non-renal (n = 157)	Cluster 4 Renal without high CRP (n = 157)
Female/male	22/20	32/39	96/61	93/64
Ages (years)	63 (59–73)	73 (65–79)	73 (64–78)	73 (64–78)
GPA (%)	34 (81)	19 (27)	23 (15)	10 (6)
MPA (%)	5 (12)	49 (69)	84 (53)	132 (84)
Unclassifiable (%)	3 (7)	3 (4)	50 (32)	15 (10)
MPO + ANCA (%)	3 (7)	71 (100)	150 (96)	156 (99)
PR3-ANCA (%)	33 (79)	6 (9)	5 (3)	3 (2)
Serum creatinine (mg/dL)	0.8 (0.6–1.4)	2.9 (1.6–4.6)	0.76 (0.62–1.0)	2.4 (1.3–4.5)
< 1.3 (%)	30 (71)	8 (11)	140 (89)	38 (24)
≥ 1.3, < 5.5 (%)	11 (26)	47 (66)	17 (11)	92 (59)
≥ 5.5 (%)	1 (2)	16 (23)	0	27 (17)
CRP (mg/dL)	3.9 (1–11)	13 (11–17)	9.6 (6.1–13)	1.6 (0.2–5.1)
< 2.6 (%)	14 (33)	0	7 (4)	99 (63)
≥ 2.6, ≤ 10 (%)	15 (36)	10 (14)	74 (47)	56 (36)
> 10 (%)	13 (31)	61 (86)	76 (48)	2 (1)
ILD (%)	6 (14)	31 (44)	86 (55)	62 (39)
General (%)	26 (62)	60 (85)	140 (89)	58 (37)
Cutaneous (%)	5 (12)	12 (17)	32 (20)	10 (6)
Mucous membranes/eyes (%)	18 (43)	8 (11)	19 (12)	5 (3)
Ear nose and throat (%)	34 (81)	23 (32)	19 (12)	13 (8)
Chest (%)	18 (43)	49 (69)	56 (36)	49 (31)
Cardiovascular (%)	3 (7)	14 (20)	3 (2)	14 (9)
Abdominal (%)	1 (2)	2 (3)	1 (1)	0
Renal (%)	24 (57)	71 (100)	105 (67)	148 (94)
Nervous system (%)	13 (31)	27 (38)	85 (54)	11 (7)

Values expressed as the number of patients or median (interquartile).

*CRP* C-reactive protein, *GPA* granulomatosis with polyangiitis, *ILD* interstitial lung disease, *MPA* microscopic polyangiitis, *MPO-ANCA* myeloperoxidase-antineutrophil cytoplasmic antibody, *PR3-ANCA* proteinase-3-antineutrophil cytoplasmic antibody.

**Figure 4:**
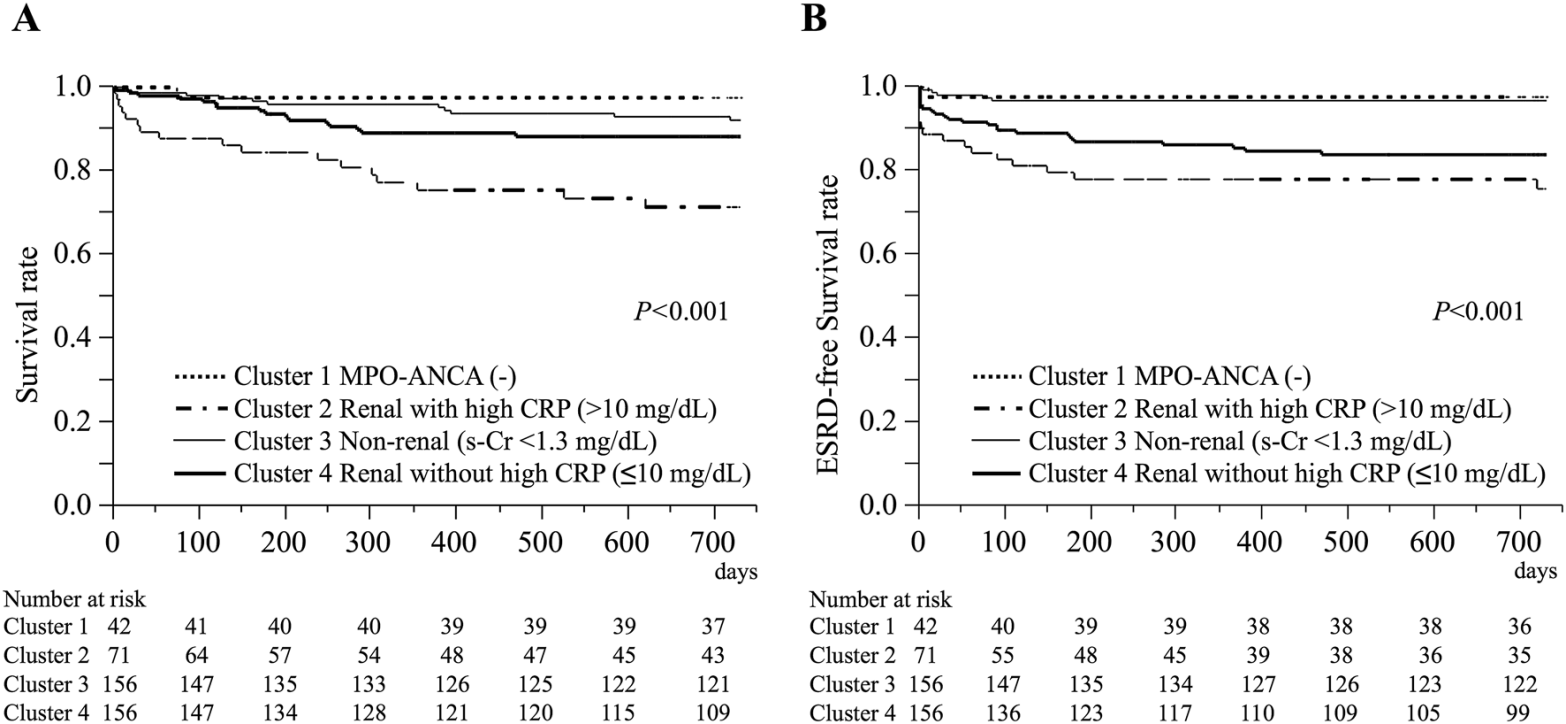
Overall survival and ESRD-free survival rates according to clusters of model 2. (**A**) Overall survival rates and (**B**) ESRD-free survival rates. Analysis was performed using a log-rank test. Cluster 2 showed a worse survival rate compared to Cluster 1 (*P* = 0.0024) and Cluster 3 (*P* = 0.0003). Cluster 2 exhibited worse ESRD-free survival rate compared to Cluster 1 (*P* < 0.0001) and Cluster 3 (*P* < 0.0001). One patient in Cluster 3 and one patient in Cluster 4 were excluded from these analyses because of the missing follow-up data. *ESRD* end-stage renal disease, *CRP* C-reactive protein, *MPO-ANCA* myeloperoxidase-antineutrophil cytoplasmic antibody, *s-Cr* serum creatinine.

The overall survival rates differed significantly across the four clusters (*P* < 0.001, Fig. 4A). Cluster 2 (MPO-ANCA-positive renal patients with high CRP) showed a worse survival rate compared to Cluster 1 (MPO-ANCA-negative, *P* = 0.002) and Cluster 3 (MPO-ANCA-positive non-renal patients, *P* < 0.001, Fig. 4A). The ESRD-free survival rate also differed significantly among the 4 clusters (*P* < 0.001, Fig. 4B), and Cluster 2 exhibited a worse ESRD-free survival rate compared to Cluster 1 (*P* < 0.001) and Cluster 3 (*P* < 0.001). The cumulative remission rates did not differ (*P* = 0.173, Supplementary Fig. S5A), but relapse-free survival was significantly different across the four clusters of model 2 (*P* = 0.023, Supplementary Fig. S5B). Cluster 2 and Cluster 3 exhibited worse relapse-free survival rates compared with Cluster 1 (*P* = 0.006 and *P* = 0.009, respectively, Supplementary Fig. S5B). When comparing MPO-ANCA negative patients (Cluster 1) with other MPO-ANCA positive clusters, the remission and overall survival rates showed no statistical difference (*P* = 0.585 and *P* = 0.065) while ESRD-free survival and relapse-free survival rates were better in MPO-ANCA negative cluster compared to other MPO-ANCA positive clusters (*P* = 0.020 and *P* = 0.014).

### Association between CRP and clinical symptoms

We explored the clinical symptoms associated with the CRP level using BVAS items and ILD. Univariate regression analysis revealed that ‘fever’, ‘weight loss’, ‘myalgia’, ‘arthralgia/arthritis’, ‘conductive hearing loss’, ‘pleural effusion/pleurisy’, ‘infiltrate’, ‘massive hemoptysis/alveolar hemorrhage’, ‘sensory peripheral neuropathy’, and ‘mononeuritis multiplex’ associated with CRP levels. A multiple linear regression analysis exhibited that fever (β coefficient (95% confidence interval) 2.1 (1.5–2.6)), myalgia (2.5 (2.0–3.1)), massive hemoptysis/alveolar hemorrhage (1.3 (0.4–2.2)), and mononeuritis multiplex (1.2 (0.5–2.0)) independently associated with CRP levels (Supplementary Table S5). Renal symptoms showed no association with CRP levels.

## Discussion

This is the first study that classified Japanese AAV patients by cluster analysis and evaluated the prognosis among the determined clusters. In the first model, seven distinct clinical clusters were identified: ENT, cutaneous, non-renal, and renal in MPO-ANCA-positive patients; PR3-ANCA-positive patients; and ANCA-negative patients. Moreover, clusters separated on the basis of CRP and s-Cr levels predicted prognosis with respect to overall, ESRD-free, and relapse-free survival rates. Further, this is the first study to evaluate the symptoms which contribute to the CRP level, and the analysis suggested that the CRP level represented general, pulmonary, and neural symptoms but not renal symptoms.

Although the ANCA phenotype and the ENT, cutaneous, and renal symptoms are universal and specific features of AAV, it is possible that the priority for each feature in the classification of AAV is different among different ethnicities or regions. In this study, PR3-ANCA-positive and ANCA-negative patients were first separated. A previous report from a Western country demonstrated that PR3-ANCA positivity was used to classify patients with GPA and MPA into two clusters in the final branch of classification^5^. In that study, 56% of patients was PR3-ANCA-positive and 32% was MPO-ANCA-positive, while, in our study, 89% was MPO-ANCA-positive but only 11% was PR3-ANCA-positive. Although the importance of the ANCA phenotype in our study was consistent with that in the previous study, this apparent difference in ANCA positivity might determine the distinction between two clusters according to the ANCA phenotype ahead of clinical symptoms^5^. Subsequently, patients with ENT symptoms were classified among MPO-ANCA-positive patients. Although ENT symptoms are generally regarded as surrogate markers for GPA^11^, which is characterized by PR3-ANCA, there are several reports regarding MPO-ANCA-positive GPA, in which the majority of patients show ENT symptoms, consistent with our results^17,18^. Recently, the notion of otitis media with AAV (OMAAV) irrespective of the ANCA phenotype has emerged^19^, which frequently accompanies facial palsy and hypertrophic pachymeningitis. Thus, ENT symptoms may be a surrogate clinical marker for AAV even in MPO-ANCA-positive AAV. After classification based on ENT symptoms, patients with cutaneous symptoms were identified. This cluster exhibited various organ involvements except for ENT symptoms (Table 1). According to the previous report^20^, cutaneous vasculitis rarely preceded the renal and pulmonary symptoms in patients with MPA. Cutaneous vasculitis was also reported to be associated with general, renal, pulmonary, and nervous system symptoms^20,21^. Indeed, the cutaneous cluster showed a tendency toward worse overall survival in our study. Therefore, patients with cutaneous symptoms should be carefully evaluated for systemic organ involvements. Finally, clusters with and without renal symptoms were separated. In our previous report, using a different cohort, renal vasculitis was observed in 75% of MPO-ANCA-positive AAV^22^. In the present study, we have been able to confirm that renal symptoms are specific for MPO-ANCA-positive MPA and GPA.

s-Cr and CRP levels could be good biomarkers for the prediction of prognosis. In the present study, patients with ≥ 1.3 mg/dL (120 μmol/L) of s-Cr levels showed poor overall and ESRD-free survival. Patients with < 1.3 mg/dL of s-Cr levels were categorized as having the localized type or early systemic type of AAV^15^. The BVAS has categorized s-Cr levels into 3 groups: 1.4 mg/dL (125 μmol/L)–2.8 mg/dL (249 μmol/L), 2.8 mg/dL (250 μmol/L)–5.6 mg/dL (499 μmol/L), and ≥ 5.6 mg/dL (500 μmol/L)^12^. The 1996 Five-Factor Score also included s-Cr levels of > 1.6 mg/dL as the indicator for renal insufficiency^23^. Although previous reports have shown that better renal function at diagnosis is associated with improved survival and renal outcome^24–26^, the presence of renal insufficiency might be more important than the severity of renal insufficiency for the prediction of prognosis in patients with AAV. In addition to the s-Cr levels, the CRP levels could differentiate the prognosis of AAV. Previous reports have showed that an increase in CRP levels before treatment of AAV was associated with relapse and mortality^9,10^. Though the appropriate cut-off of CRP for the prediction of AAV outcomes had not been determined, we have reported the utility of the Japanese RPGN clinical grading system for predicting prognosis, which consists of age, s-Cr levels, lung complication, and CRP levels where the CRP levels were separated by 2.6 mg/dL and 10.0 mg/dL^8^. In the present study, we were able to validate these cut-offs of CRP levels. Further studies are needed to confirm the roles of s-Cr and CRP levels as biomarkers for prognosis in patients with AAV.

CRP is a surrogate marker for important organ involvements, except for renal symptoms. The CRP levels have not been observed to increase in patients with glomerulonephritis including IgA nephropathy, membranous nephropathy, and minimal change disease compared to that in controls^27^. Consistent with these previous observations, the CRP levels associated with fever, myalgia, and pulmonary and neurological vasculitis but not with renal symptoms in the present study. Although several previous reports have described that CRP could be a biomarker in the patients with systemic lupus erythematosus or AAV with renal involvements^28,29^, CRP levels may be related to organ involvements other than the kidney in the patients with AAV. Therefore, it could be rational and relevant to perform a combined evaluation of s-Cr and CRP as indicators of the severity of renal and non-renal symptoms, respectively, for predicting prognosis in patients with AAV. Importantly, these biomarkers are modifiable by treatment. Hence, future research should elucidate whether improvements in CRP and/or s-Cr levels by treatments can predict prognosis.

Several limitations of this study should be acknowledged. First, we could not validate our classification models. Different populations are required for the validation of our results, which will be conducted in the near future. Second, MPO-ANCA-positive MPA is dominant in the population of Japan in contrast to the dominance of PR3-ANCA in the Western population. However, this point might be strength of our study because new insights for clinical characteristics were provided by different cluster analyses in Western populations. Third, AAV was diagnosed clinically by the site investigators, though all patients were fulfilled the criteria for primary systemic vasculitis and classified by the EMEA algorithm. Forth, the treatment strategy was decided at the discretion of each attending physician; therefore, it is possible that patients with higher s-Cr levels were treated intensively, leading to underestimation of outcomes. Nevertheless, patients with higher s-Cr levels showed worse overall and renal survival, supporting the relevance of renal vasculitis in AAV. Fifth, BVAS items with low prevalence were excluded from the analysis of the association with CRP levels. Among the excluded items, congestive cardiac failure, peritonitis, bloody diarrhea, and ischemic abdominal pain have been reportedly related to survival in patients with AAV^30^. Therefore, careful workup for these cardiovascular and abdominal involvements may be necessary, regardless of CRP levels.

## Conclusions

In summary, we have identified novel clusters of AAV among Japanese patients. We have also identified s-Cr level, CRP level, and MPO-ANCA negativity as prognostic biomarkers for overall, ESRD-free, and relapse-free survival. Further, we found that CRP was associated with non-renal symptoms such as general, pulmonary and nervous system symptoms but not with renal symptoms.

### Supplementary Information

Supplementary Information.

Supplementary Figure S1.

Supplementary Figure S2.

Supplementary Figure S3.

Supplementary Figure S4.

Supplementary Figure S5.

## Data Availability

Data is available through the corresponding author on reasonable request.

## Appendix

Japan Research Committee of the Ministry of Health, Labour, and Welfare for Intractable Vasculitis (JPVAS) and Research Committee of Intractable Renal Disease of the Ministry of Health, Labour, and Welfare of Japan: In addition to the authors, the following investigators and institutions participated in this study: Department of Human Resource Development of Dialysis Therapy for Kidney Disease, Okayama University Graduate School of Medicine, Dentistry and Pharmaceutical Sciences (Hitoshi Sugiyama); Department of Nephrology, Internal Medicine, Nagoya University Graduate School of Medicine (Seiichi Matsuo); Department of the Control for Rheumatic Diseases, Graduate School of Medicine, Kyoto University (Takao Fujii); Department of Nephrology, Fujita Health University School of Medicine, (Yukio Yuzawa); Department of Nephrology, Internal Medicine, Nagoya University Graduate School of Medicine (Naotake Tsuboi); Department of Nephrology and Dialysis, Kitano Hospital, Tazuke Kofukai Medical Research Institute (Eri Muso); Department of Nephrology, Faculty of Medicine, University of Tsukuba (Joichi Usui); Department of Clinical Pathology and Immunology, Kobe University Graduate School of Medicine (Shunichi Kumagai); Department of Medicine and Rheumatology, Tokyo Metropolitan Geriatric Hospital (Takahiko Sugihara); Department of Rheumatology, Shimane University Faculty of Medicine (Yohko Murakawa); Division of Nephrology and Rheumatology, Department of Internal Medicine Aichi Medical University School of Medicine (Shogo Banno); Department of Hematology, Clinical Immunology and Infectious Diseases, Ehime University Graduate School of Medicine (Hitoshi Hasegawa); Division of Nephrology, Department of Internal Medicine, Jichi Medical University (Wako Yumura); Department of Cardiovascular Medicine, Kyoto Prefectural University School of Medicine (Hiroaki Matsubara); Division of Nephrology, Tokyo Medical University Hachioji Medical Center (Masaharu Yoshida);; Division of Immunology and Rheumatology, Department of Internal Medicine 3, Hamamatsu University School of Medicine (Noriyoshi Ogawa); Department of Hematology, Oncology, Nephrology, and Rheumatology, Akita University Graduate School of Medicine (Atsushi Komatsuda); Department of Rheumatology, Niigata Rheumatic Center (Satoshi Ito); Department of Immunology and Rheumatology, Division of Advanced Preventive Medical Sciences, Nagasaki University Graduate School of Biomedical Sciences (Atsushi Kawakami); Department of Nephrology, Iwate Prefectural Central Hospital (Izaya Nakaya); Division of Nephrology and Rheumatology, Department of Internal Medicine, Fukuoka University School of Medicine (Takao Saito); Shimane University, Faculty of Medicine, Division of Nephrology (Takafumi Ito); Department of Hemodialysis and Apheresis, Yokohama City University Medical Center (Nobuhito Hirawa); Center for Rheumatology, Okayama Saiseikai General Hospital (Masahiro Yamamura); Department of Medical Technology, School of Health Sciences, Faculty of Medicine, Niigata University (Masaaki Nakano); Department of Medicine, Kidney Center, Tokyo Women’s Medical University (Kosaku Nitta); Division of Nephrology and Hypertension, Kashiwa Hospital, Jikei University (Makoto Ogura); Department of Respiratory Medicine, Allergy and Clinical Immunology, Nagoya City University Graduate School of Medical Sciences (Taio Naniwa); Division of Rheumatology and Allergology, Department of Internal Medicine, St. Marianna University School of Medicine (Shoichi Ozaki); Department of Nephrology and Endocrinology, Graduate School of Medicine, The University of Tokyo (Junichi Hirahashi); Division of Kidney and Hypertension, Department of Internal Medicine, Jikei University School of Medicine (Tatsuo Hosoya); Department of Nephrology and Laboratory Medicine, Faculty of Medicine, Institute of Medical, Pharmaceutical and Health Sciences, Kanazawa University (Takashi Wada); Division of Nephrology, Department of Internal Medicine, Juntendo University Faculty of Medicine (Satoshi Horikoshi); Institute of Rheumatology, Tokyo Women’s Medical University (Yasushi Kawaguchi); Division of Clinical Immunology, Graduate School of Comprehensive Human Sciences, University of Tsukuba (Taichi Hayashi); Department of Nephrology, Hypertension, Diabetology, Endocrinology and Metabolic, Fukushima Medical University (Tsuyoshi Watanabe); Department of Nephrology, Japanese Red Cross Nagoya Daini Hospital (Daijo Inaguma); Department of Integrated Therapy for Chronic Kidney Disease, Kyushu University (Kazuhiko Tsuruya); Niigata Prefectural Shibata Hospital (Noriyuki Homma); Division of Rheumatology, Department of Internal Medicine, Keio University School of Medicine (Tsutomu Takeuchi); Division of Cardiology, Nephrology, Pulmonology and Neurology, Department of Internal Medicine, Asahikawa Medical University (Naoki Nakagawa); Kurobe City Hospital (Shinichi Takeda); National Fukuoka Higashi Medical Center (Ritsuko Katafuchi); Division of Nephrology, Department of Medicine, Faculty of Medical Sciences, University of Fukui (Masayuki Iwano); Tokyo Medical University Ibaraki Medical Center (Masaki Kobayashi); Division of Rheumatology and Allergology, Department of Internal Medicine, St. Marianna University School of Medicine (Hidehiro Yamada).

## Abbreviations

AAV: Antineutrophil cytoplasmic antibody-associated vasculitis
ANCA: Antineutrophil cytoplasmic antibody
BVAS: Birmingham vasculitis activity score
CRP: C-reactive protein
EGPA: Eosinophilic granulomatosis with polyangiitis
ENT: Ear, nose and throat; ILD
ESRD: End-stage renal disease
EULAR: European League against Rheumatism
EUVAS: European Vasculitis Study Group
GPA: Granulomatosis with polyangiitis
ILD: Interstitial lung disease
IQR: Interquartile
MPA: Microscopic polyangiitis
MPO: Myeloperoxidase
OMAAV: Otitis media with AAV
PCA: Principal component analysis
PR3: Proteinase-3
RemIT-JAV: Remission induction therapy in Japanese patients with AAV
RemIT-JAV-RPGN: Remission induction therapy in Japanese patients with AAV and rapidly progressive glomerulonephritis
s-Cr: Serum creatinine

## References

[1] Jennette J, Falk R, Bacon P, Basu N, Cid M, Ferrario F, Flores-Suarez L, Gross W, Guillevin L, Hagen E (2013). 2012 revised international Chapel Hill consensus conference nomenclature of vasculitides. Arthritis Rheum..

[2] Ball GV, Fessler BJ, Bridges SL (2014). Chapter 6: Autoantibodies in vasculitis, Oxford textbook of vasculitis.

[3] Sada KE, Yamamura M, Harigai M, Fujii T, Dobashi H, Takasaki Y, Ito S, Yamada H, Wada T, Hirahashi J (2014). Classification and characteristics of Japanese patients with antineutrophil cytoplasmic antibody-associated vasculitis in a nationwide, prospective, inception cohort study. Arthrit. Res. Therapy.

[4] Lyons PA, Rayner TF, Trivedi S, Holle JU, Watts RA, Jayne DR, Baslund B, Brenchley P, Bruchfeld A, Chaudhry AN (2012). Genetically distinct subsets within ANCA-associated vasculitis. N. Engl. J. Med..

[5] Mahr A, Katsahian S, Varet H, Guillevin L, Hagen EC, Hoglund P, Merkel PA, Pagnoux C, Rasmussen N, Westman K (2013). Revisiting the classification of clinical phenotypes of anti-neutrophil cytoplasmic antibody-associated vasculitis: A cluster analysis. Ann. Rheum. Dis..

[6] Sada KE, Yamamura M, Harigai M, Fujii T, Takasaki Y, Amano K, Fujimoto S, Muso E, Murakawa Y, Arimura Y (2015). Different responses to treatment across classified diseases and severities in Japanese patients with microscopic polyangiitis and granulomatosis with polyangiitis: A nationwide prospective inception cohort study. Arthrit. Res. Therapy.

[7] Reinhold-Keller E, Herlyn K, Wagner-Bastmeyer R, Gross WL (2005). Stable incidence of primary systemic vasculitides over five years: Results from the German vasculitis register. Arthritis Rheum..

[8] Sada KE, Harigai M, Amano K, Atsumi T, Fujimoto S, Yuzawa Y, Takasaki Y, Banno S, Sugihara T, Kobayashi M (2016). Comparison of severity classification in Japanese patients with antineutrophil cytoplasmic antibody-associated vasculitis in a nationwide, prospective, inception cohort study. Mod. Rheumatol..

[9] Slot MC, Tervaert JW, Franssen CF, Stegeman CA (2003). Renal survival and prognostic factors in patients with PR3-ANCA associated vasculitis with renal involvement. Kidney Int..

[10] Kalsch AI, Csernok E, Munch D, Birck R, Yard BA, Gross W, Kalsch T, Schmitt WH (2010). Use of highly sensitive C-reactive protein for followup of Wegener's granulomatosis. J. Rheumatol..

[11] Watts R, Lane S, Hanslik T, Hauser T, Hellmich B, Koldingsnes W, Mahr A, Segelmark M, Cohen-Tervaert JW, Scott D (2007). Development and validation of a consensus methodology for the classification of the ANCA-associated vasculitides and polyarteritis nodosa for epidemiological studies. Ann. Rheum. Dis..

[12] Luqmani RA, Bacon PA, Moots RJ, Janssen BA, Pall A, Emery P, Savage C, Adu D (1994). Birmingham vasculitis activity score (BVAS) in systemic necrotizing vasculitis. QJM.

[13] Jones RB, Hiemstra TF, Ballarin J, Blockmans DE, Brogan P, Bruchfeld A, Cid MC, Dahlsveen K, de Zoysa J, Espigol-Frigolé G (2019). Mycophenolate mofetil versus cyclophosphamide for remission induction in ANCA-associated vasculitis: A randomised, non-inferiority trial. Ann. Rheum. Dis..

[14] Mukhtyar C, Guillevin L, Cid MC, Dasgupta B, de Groot K, Gross W, Hauser T, Hellmich B, Jayne D, Kallenberg CG (2009). EULAR recommendations for the management of primary small and medium vessel vasculitis. Ann. Rheum. Dis..

[15] Hellmich B, Flossmann O, Gross WL, Bacon P, Cohen-Tervaert JW, Guillevin L, Jayne D, Mahr A, Merkel PA, Raspe H (2007). EULAR recommendations for conducting clinical studies and/or clinical trials in systemic vasculitis: Focus on anti-neutrophil cytoplasm antibody-associated vasculitis. Ann. Rheum. Dis..

[16] Koyama A, Yamagata K, Makino H, Arimura Y, Wada T, Nitta K, Nihei H, Muso E, Taguma Y, Shigematsu H (2009). A nationwide survey of rapidly progressive glomerulonephritis in Japan: Etiology, prognosis and treatment diversity. Clin. Exp. Nephrol..

[17] Miloslavsky EM, Lu N, Unizony S, Choi HK, Merkel PA, Seo P, Spiera R, Langford CA, Hoffman GS, Kallenberg CG (2016). Myeloperoxidase-antineutrophil cytoplasmic antibody (ANCA)-positive and ANCA-negative patients with granulomatosis with polyangiitis (Wegener's): Distinct patient subsets. Arthritis Rheumatol..

[18] Ono N, Niiro H, Ueda A, Sawabe T, Nishizaka H, Furugo I, Yoshizawa S, Yoshizawa S, Tsukamoto H, Kiyohara C (2015). Characteristics of MPO-ANCA-positive granulomatosis with polyangiitis: A retrospective multi-center study in Japan. Rheumatol. Int..

[19] Harabuchi Y, Kishibe K, Tateyama K, Morita Y, Yoshida N, Kunimoto Y, Matsui T, Sakaguchi H, Okada M, Watanabe T (2017). Clinical features and treatment outcomes of otitis media with antineutrophil cytoplasmic antibody (ANCA)-associated vasculitis (OMAAV): A retrospective analysis of 235 patients from a nationwide survey in Japan. Mod. Rheumatol..

[20] Niiyama S, Amoh Y, Tomita M, Katsuoka K (2008). Dermatological manifestations associated with microscopic polyangiitis. Rheumatol Int.

[21] Kluger N, Pagnoux C, Guillevin L, Frances C (2008). Comparison of cutaneous manifestations in systemic polyarteritis nodosa and microscopic polyangiitis. Br. J. Dermatol..

[22] Ozaki S, Atsumi T, Hayashi T, Ishizu A, Kobayashi S, Kumagai S, Kurihara Y, Kurokawa MS, Makino H, Nagafuchi H (2012). Severity-based treatment for Japanese patients with MPO-ANCA-associated vasculitis: The JMAAV study. Mod. Rheumatol..

[23] Guillevin L, Lhote F, Gayraud M, Cohen P, Jarrousse B, Lortholary O, Thibult N, Casassus P (1996). Prognostic factors in polyarteritis nodosa and Churg-Strauss syndrome. A prospective study in 342 patients. Medicine (Baltimore).

[24] Hogan SL, Nachman PH, Wilkman AS, Jennette JC, Falk RJ (1996). Prognostic markers in patients with antineutrophil cytoplasmic autoantibody-associated microscopic polyangiitis and glomerulonephritis. J. Am. Soc. Nephrol..

[25] Westman KW, Selga D, Isberg PE, Bladstrom A, Olsson H (2003). High proteinase 3-anti-neutrophil cytoplasmic antibody (ANCA) level measured by the capture enzyme-linked immunosorbent assay method is associated with decreased patient survival in ANCA-associated vasculitis with renal involvement. J. Am. Soc. Nephrol..

[26] Booth AD, Almond MK, Burns A, Ellis P, Gaskin G, Neild GH, Plaisance M, Pusey CD, Jayne DR (2003). Outcome of ANCA-associated renal vasculitis: A 5-year retrospective study. Am. J. Kidney Dis..

[27] Baek JE, Chang JW, Min WK, Cho YM, Park JS, Kim SB (2008). Serum high-sensitivity C-reactive protein is not increased in patients with IgA nephropathy. Nephron Clin. Pract..

[28] Gaitonde S, Samols D, Kushner I (2008). C-reactive protein and systemic lupus erythematosus. Arthritis Rheum..

[29] Kronbichler A, Kerschbaum J, Gründlinger G, Leierer J, Mayer G, Rudnicki M (2016). Evaluation and validation of biomarkers in granulomatosis with polyangiitis and microscopic polyangiitis. Nephrol. Dial Transplant.

[30] Guillevin L, Pagnoux C, Seror R, Mahr A, Mouthon L, Le Toumelin P (2011). (FVSG) FVSG: The five-factor score revisited: Assessment of prognoses of systemic necrotizing vasculitides based on the French Vasculitis Study Group (FVSG) cohort. Medicine (Baltimore).

