# Supplementary material for: Exploratory classification of clinical phenotypes in Japanese patients with antineutrophil cytoplasmic antibody-associated vasculitis using cluster analysis
Source: Sci Rep. 2021 Mar 4;11:5223. doi: 10.1038/s41598-021-84627-6 (PMC7933174; doi:10.1038/s41598-021-84627-6)
Supplement: Supplementary file 1 — Supplementary Information. [file 41598_2021_84627_MOESM1_ESM.docx]

**Exploratory classification of clinical phenotypes in Japanese patients with** **antineutrophil cytoplasmic antibody-associated vasculitis using cluster analysis**

Haruki Watanabe^1^, MD, PhD, Ken-ei Sada^1^*, MD, PhD, Masayoshi Harigai^2^, MD, PhD, Koichi Amano^3^, MD, PhD, Hiroaki Dobashi^4^, MD, PhD, Yoshinari Takasaki^5^, MD, PhD, Shouichi Fujimoto^6^, MD, PhD, Tatsuya Atsumi^7^, MD, PhD, Kunihiro Yamagata^8^, MD, PhD, Sakae Homma^9^, MD, PhD, Yoshihiro Arimura^10, 11^, MD, PhD, and Hirofumi Makino^12^, MD, PhD for Research Committee of Intractable Vasculitis Syndrome (JPVAS) and Research Committee of Intractable Renal Disease of the Ministry of Health, Labour, and Welfare of Japan.

^1^Department of Nephrology, Rheumatology, Endocrinology and Metabolism, Okayama University Graduate School of Medicine, Dentistry and Pharmaceutical Sciences, 2-5-1 Shikata-cho, Kita-ku, Okayama 700-8558, Japan

^2^Department of Rheumatology, Tokyo Women's Medical University School of Medicine, Tokyo, Japan

^3^Department of Rheumatology and Clinical Immunology, Saitama Medical Center, Saitama Medical University, Kawagoe, Japan

^4^Division of Hematology, Rheumatology and Respiratory Medicine, Department of Internal Medicine, Faculty of Medicine, Kagawa University, Kita-gun, Miki-cho, Japan

^5^Department of Internal Medicine and Rheumatology, Juntendo University School of Medicine, Tokyo, Japan

^6^Department of Hemovascular Medicine and Artificial Organs, Faculty of Medicine, University of Miyazaki, Miyazaki, Japan

^7^Department of Rheumatology, Endocrinology and Nephrology, Faculty of Medicine and

Graduate School of Medicine, Hokkaido University, Sapporo, Japan

^8^Department of Nephrology, Faculty of Medicine, University of Tsukuba, Ibaraki, Japan

^9^ Department of Advanced and Integrated Interstitial Lung Diseases Research, School of Medicine, Toho University, Tokyo, Japan

^10^ Department of Nephrology and Rheumatology, Kyorin University School of Medicine, Tokyo, Japan

^11^Kichijoji Asahi Hosipital

^12^Okayama University, Okayama, Japan

**Supplementary figure 1**. **Flow chart showing the enrollment of patients**

**From the 321 patients from the RemIT-JAV-RPGN and 156 from the RemIT-JAV, patients with EGPA and patients whose PR3-ANCA results were unavailable at diagnosis were excluded.**

**EGPA, eosinophilic granulomatosis with polyangiitis; RemIT-JAV-RPGN, Remission Induction Therapy in Japanese Patients With ANCA-Associated Vasculitides and Rapidly Progressive Glomerulonephritis; PR3-ANCA, proteinase-3-antineutrophil cytoplasmic antibody.**

**Supplementary figure 2**. Dendrograms for cluster model 1 for antineutrophil cytoplasmic antibody-associated vasculitis

The dendrogram shows the clustering process of model 1 resulting in seven clusters. Cluster 1 was PR3-ANCA-positive, and Cluster 2 was characterized by ANCA negativity. All patients in the other five clusters were MPO-ANCA-positive. Cluster 3 was characterized by ENT symptoms, Cluster 4 by cutaneous symptoms, Cluster 5 by renal symptoms, Cluster 6 by non-renal symptoms, and Cluster 7 by ENT and cutaneous symptoms.

ENT, ear, nose and throat; MPO-ANCA, myeloperoxidase-antineutrophil cytoplasmic antibody; PR3-ANCA, proteinase-3-antineutrophil cytoplasmic antibody.

**Supplementary figure 3.** Overall and ESRD-free survival rates according to clusters of model 1

(A) Overall survival rates and (B) ESRD-free survival rates.

Analysis was performed using a log-rank test. ESRD-free survival rate differed significantly across the seven clusters, but no significant difference was found between any two clusters. One patient in Cluster 5 and one patient in Cluster 6 were excluded from these analyses because of the missing follow-up data.

ESRD, end-stage renal disease.

**Supplementary figure 4.** Remission and relapse-free survival rates according to clusters of model 1

(A) Remission rates and (B) relapse-free survival rates.

Analysis performed using a log-rank test. The evaluation of relapse began from the day that BVAS remission was achieved, and day 0 of the right panel means the day when the remission has been achieved accordingly. One patient in Cluster 5 and one patient in Cluster 6 were excluded from these analyses because of the missing follow-up data.

BVAS, Birmingham Vasculitis Activity Score.

**Supplementary figure 5.** Remission and relapse-free survival rates according to clusters of model 2

(A) Remission rates and (B) relapse-free survival rates.

Analysis was performed using a log-rank test. The evaluation of relapse began from the day that BVAS remission was achieved, and day 0 of the right panel means the day when the remission has been achieved accordingly. The relapse-free survival was significantly different across the four clusters of model 2, and Cluster 2 and Cluster 3 exhibited worse relapse-free survival rates compared with Cluster 1 (*P*=0.0058 and *P*=0.0087, respectively). One patient in Cluster 3 and one patient in Cluster 4 were excluded from these analyses because of the missing follow-up data.

BVAS, Birmingham Vasculitis Activity Score, CRP, C-reactive protein; MPO-ANCA, myeloperoxidase-antineutrophil cytoplasmic antibody; s-Cr, serum creatinine.

**Supplementary table 1. Prevalence of each BVAS item at enrolment**

| **BVAS item** |  | **Prevalence (%)** |
| --- | --- | --- |
| 1. General | **Myalgia** | **31.0** |
|  | **Arthralgia or arthritis** | **32.0** |
|  | **Fever ≥38°C** | **32.0** |
|  | **Weight loss ≥2 kg** | **31.0** |
| 2. Cutaneous | Infarct | 0.7 |
|  | **Purpura** | **10.0** |
|  | Ulcer | 1.9 |
|  | Gangrene | 0.9 |
|  | Other skin vasculitis | 4.0 |
| 3. Mucous membranes / eyes | Mouth ulcers / granulomata | 1.4 |
|  | Genital ulcers | 0 |
|  | Adnexal inflammation | 0 |
|  | Significant proptosis | 0.7 |
|  | Scleritis / Episcleritis | 4.7 |
|  | Conjunctivitis / Blepharitis / Keratitis | 2.3 |
|  | Blurred vision | 1.6 |
|  | Sudden visual loss | 1.2 |
|  | Uveitis | 0.2 |
|  | Retinal changes (vasculitis / thrombosis / exudate / hemorrhage) | 2.8 |
| 4. Ear, nose and throat | **Bloody nasal discharge / crusts / ulcers / granulomata** | **7.7** |
|  | **Paranasal sinus involvement** | **15.0** |
|  | Subglottic stenosis | 0.7 |
|  | **Conductive hearing loss** | **6.8** |
|  | **Sensorineural hearing loss** | **7.0** |
| 5. Chest | Wheeze | 2.6 |
|  | **Nodules or cavities** | **8.9** |
|  | **Pleural effusion / pleurisy** | **16.0** |
|  | **Infiltrate** | **22.0** |
|  | Endobronchial involvement | 2.3 |
|  | **Massive hemoptysis / alveolar hemorrhage** | **8.9** |
|  | Respiratory failure | 1.4 |
| 6. Cardiovascular | Loss of pulses | 1.2 |
|  | Valvular heart disease | 1.6 |
|  | Pericarditis | 0.7 |
|  | Ischemic cardiac pain | 0 |
|  | Cardiomyopathy | 0.9 |
|  | Congestive cardiac failure | 4.5 |
| 7. Abdominal | Peritonitis | 0 |
|  | Bloody diarrhea | 0.5 |
|  | Ischemic abdominal pain | 0.7 |
| 8. Renal | **Hypertension** | **18.0** |
|  | **Proteinuria >1+ on urinalysis or >0.2 g/g creatinine** | **74.0** |
|  | **Haematuria ‘Moderate’ on urinalysis or ≥10 RBC per high power field** | **72.0** |
|  | **Serum creatinine >1.4 mg/dL (125 μmol/L)** | **47.0** |
|  | **>30% rise in creatinine or >25% fall in creatinine clearance or estimated glomerular filtration rate (eGFR)** | **47.0** |
| 9. Nervous system | **Headache** | **6.6** |
|  | Meningitis | 0.5 |
|  | Organic confusion | 0.5 |
|  | Seizures | 0.2 |
|  | Cerebrovascular accident | 1.2 |
|  | Spinal cord lesion | 0 |
|  | Cranial nerve palsy | 1.6 |
|  | **Sensory peripheral neuropathy** | **23.0** |
|  | **Motor mononeuritis multiplex** | **14.0** |

The items with a bold line were observed in >5% of enrolled patients and were used as candidate variables for the CRP analysis as in **Supplementary Table 5**.

BVAS, Birmingham Vasculitis Activity Score; CRP, C-reactive protein.

**Supplementary table 2. Patient characteristics**

| Female / male | 184 / 243 |
| --- | --- |
| Ages (years) | 72 (63-78) |
| GPA / MPA / Unclassifiable (%) | 86 (20) / 270 (63) / 71 (17) |
| MPO-ANCA / (%) | 380 (90) |
| PR3-ANCA (%) | 47 (11) |
| Serum creatinine (mg/dL) | 1.2 (0.7-3.4) |
| CRP (mg/dL) | 7 (2-12) |
| ILD (%) | 185 (43) |
| General (%) | 284 (67) |
| Cutaneous (%) | 59 (14) |
| Mucous membranes/eyes (%) | 50 (12) |
| Ear nose and throat (%) | 89 (21) |
| Chest (%) | 172 (40) |
| Cardiovascular (%) | 34 (8) |
| Abdominal (%) | 4 (1) |
| Renal (%) | 348 (81) |
| Nervous system (%) | 136 (312) |
| Initial glucocorticoid (mg/day)^a^ | 40 (30-50) |
| Cyclophosphamide induction therapy (%) | 152 (36) |

Values expressed as the number of patients or median (interquartile).

CRP, C-reactive protein; GPA, granulomatosis with polyangiitis; ILD, interstitial lung disease; MPA, microscopic polyangiitis; MPO-ANCA, myeloperoxidase-antineutrophil cytoplasmic antibody; PR3-ANCA, proteinase-3-antineutrophil cytoplasmic antibody.

^d^Prednisolone equivalent

**Supplementary table 3. Contribution rates of candidate variables in model 1 determined by multiple correspondence analysis**

| Variable | Contribution rates | Total contribution rates |
| --- | --- | --- |
| MPO-ANCA | 0.187 | 0.187 |
| PR3-ANCA | 0.169 | 0.356 |
| Ear, nose and throat | 0.152 | 0.508 |
| Nervous system | 0.135 | 0.643 |
| General | 0.103 | 0.746 |
| Renal | 0.056 | 0.802 |
| Cutaneous | 0.054 | 0.856 |
| Interstitial lung disease | 0.054 | 0.911 |
| Mucous membranes/eyes | 0.053 | 0.964 |
| Cardiovascular | 0.028 | 0.992 |
| Abdominal | 0.004 | 0.996 |
| Chest | 0.004 | 1.000 |

Variables above the solid line explained at least 90% of the total contribution rates. These variables were included for the cluster analysis.

MPO-ANCA, myeloperoxidase-antineutrophil cytoplasmic antibody; PR3-ANCA, proteinase-3-antineutrophil cytoplasmic antibody.

**Supplementary table 4. Contribution rates of candidate variables (including creatinine and CRP) in model 2 determined by multiple correspondence analysis**

| Variable | Contribution rates | Total contribution rates |
| --- | --- | --- |
| MPO-ANCA | 0.169 | 0.169 |
| PR3-ANCA | 0.141 | 0.310 |
| General | 0.131 | 0.441 |
| Ear, nose and throat | 0.127 | 0.567 |
| CRP | 0.116 | 0.684 |
| Nervous system | 0.083 | 0.767 |
| Creatinine | 0.069 | 0.836 |
| Mucous membranes/eyes | 0.046 | 0.882 |
| Renal | 0.043 | 0.925 |
| Interstitial lung disease | 0.041 | 0.966 |
| Cutaneous | 0.016 | 0.982 |
| Abdominal | 0.007 | 0.989 |
| Cardiovascular | 0.006 | 0.995 |
| Chest | 0.005 | 1.000 |

Variables above the solid line explained at least 90% of the total contribution rates. These variables were included for the cluster analysis.

CRP, C-reactive protein; MPO-ANCA, myeloperoxidase-antineutrophil cytoplasmic antibody; PR3-ANCA, proteinase-3-antineutrophil cytoplasmic antibody.

**Supplementary table 5. Univariate and multivariate regression analysis of the association of CRP with clinical symptoms**

|  | Univariate regression analysis | | | Multivariate regression analysis | | |
| --- | --- | --- | --- | --- | --- | --- |
|  | β coefficient | 95% CI | *P* value | β coefficient | 95% CI | *P* value |
| Fever | 2.7 | 2-3.4 | <0.0001 | 2.1 | 1.5–2.6 | <0.0001 |
| Weight loss | 4.9 | 4.3-5.5 | <0.0001 |  |  |  |
| Myalgia | 5.7 | 5.1-6.3 | <0.0001 | 2.5 | 2–3.1 | <0.0001 |
| Arthralgia/arthritis | 2.9 | 2.2-3.6 | <0.0001 |  |  |  |
| Conductive hearing loss | 3.5 | 2.3-3.7 | 0.005 |  |  |  |
| Pleural effusion/pleurisy | 2.8 | 2-3.6 | 0.001 |  |  |  |
| Infiltrate | 2.5 | 1.8-3.2 | 0.001 |  |  |  |
| Massive hemoptysis/alveolar hemorrhage | 3.2 | 2.1-4.3 | 0.003 | 1.3 | 0.43–2.2 | 0.004 |
| Sensory peripheral neuropathy | 2.1 | 1.4-2.8 | 0.005 |  |  |  |
| Mononeuritis multiplex | 3.4 | 2.5-4.3 | 0.0001 | 1.2 | 0.47–2.0 | 0.002 |

Among all 63 items of BVAS in addition to ILD, the listed items were found to be significantly related to CRP levels by univariate regression analysis. Among them, fever, myalgia, massive hemoptysis/alveolar hemorrhage, and mononeuritis multiplex were revealed to be independently associated with CRP levels by multiple linear regression analysis using stepwise backward selection to minimize the Bayesian information criterion.

BVAS, Birmingham Vasculitis Activity Score; CI, confidence interval; CRP, C-reactive protein.
