# Supplementary figures and images for: Exploratory classification of clinical phenotypes in Japanese patients with antineutrophil cytoplasmic antibody-associated vasculitis using cluster analysis
Source: Sci Rep. 2021 Mar 4;11:5223. doi: 10.1038/s41598-021-84627-6 (PMC7933174; doi:10.1038/s41598-021-84627-6)

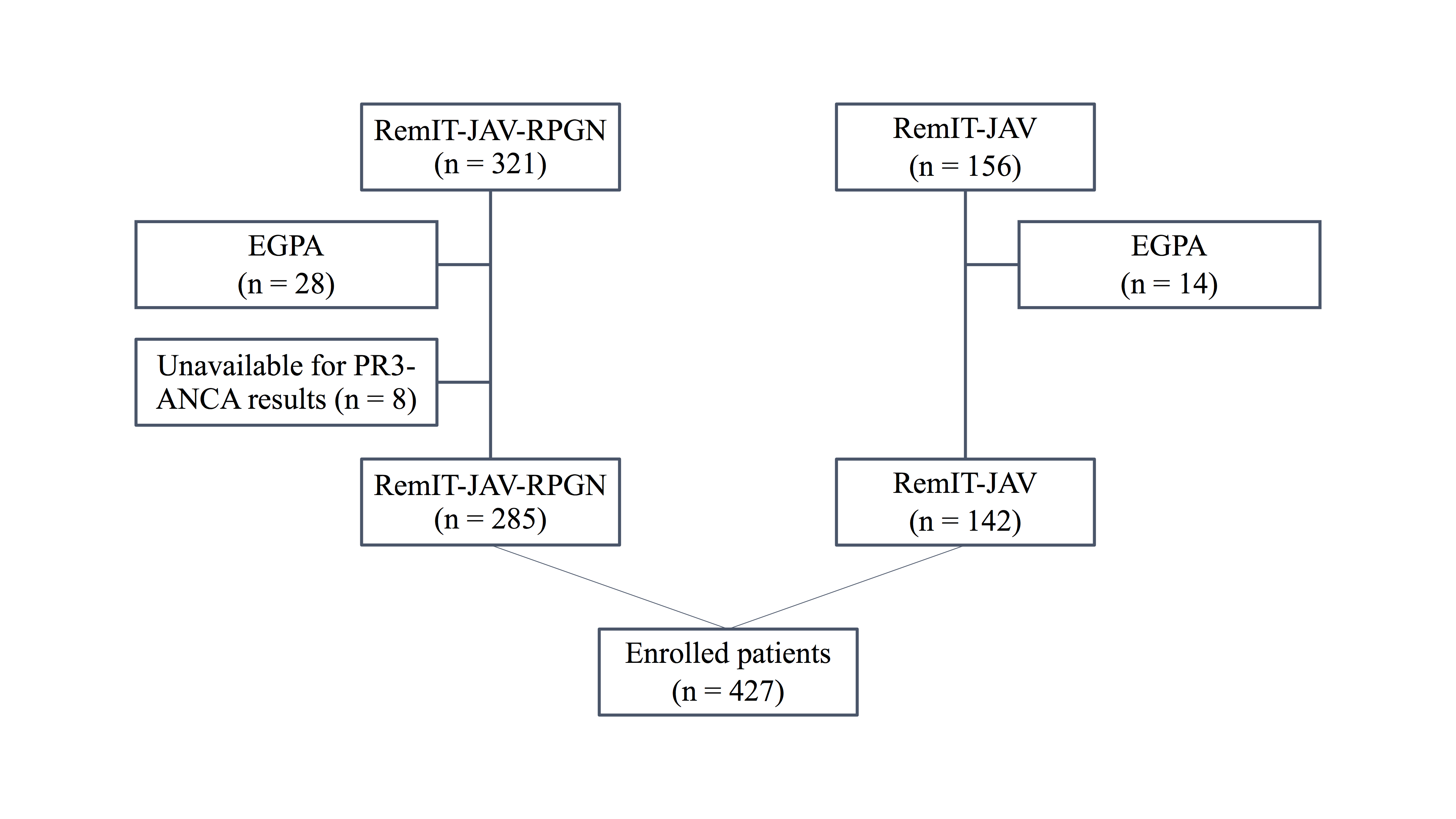

Supplement: Supplementary file 2 — Supplementary Figure S1. [file 41598_2021_84627_MOESM2_ESM.tiff]

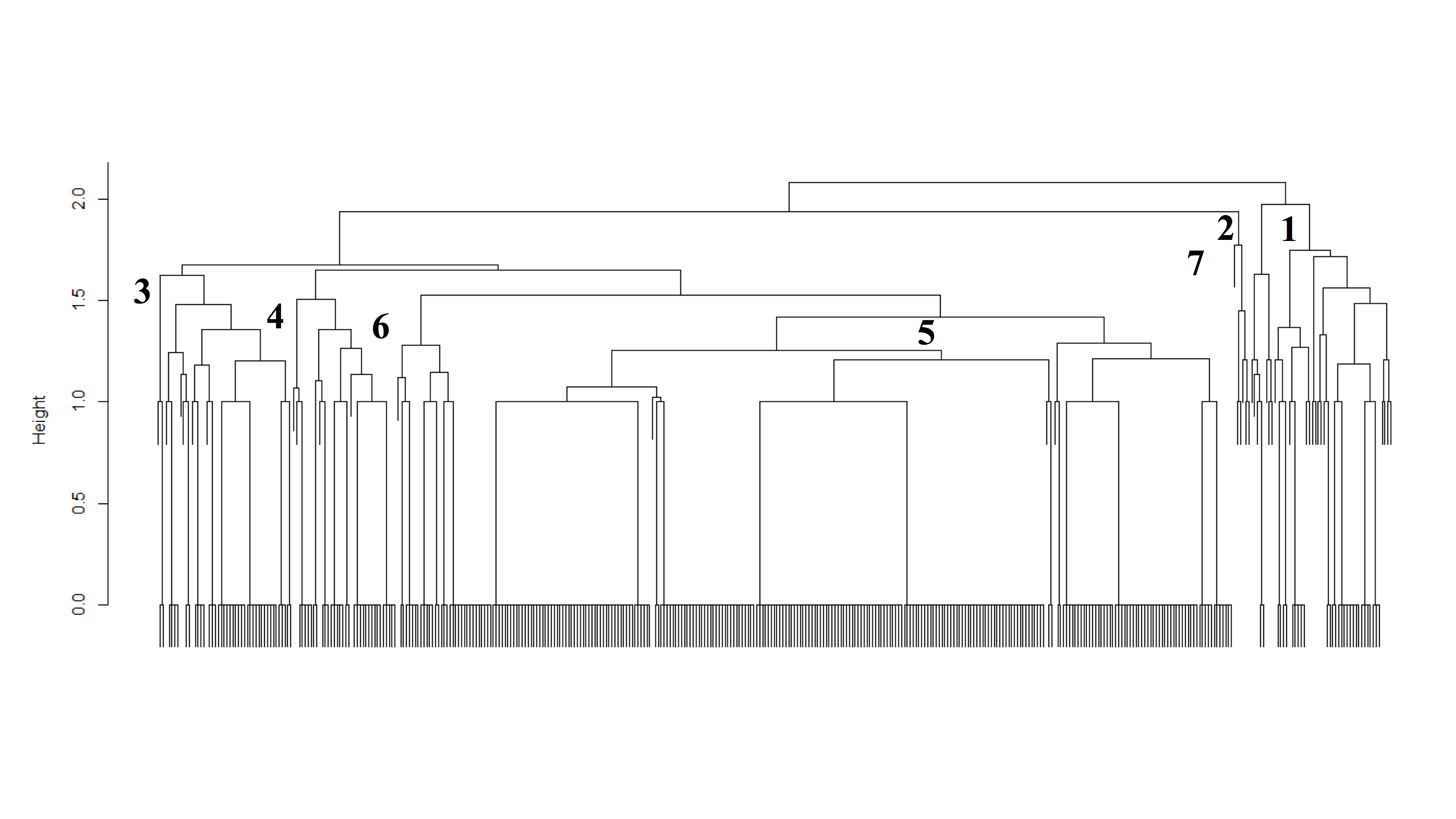

Supplement: Supplementary file 3 — Supplementary Figure S2. [file 41598_2021_84627_MOESM3_ESM.tiff]

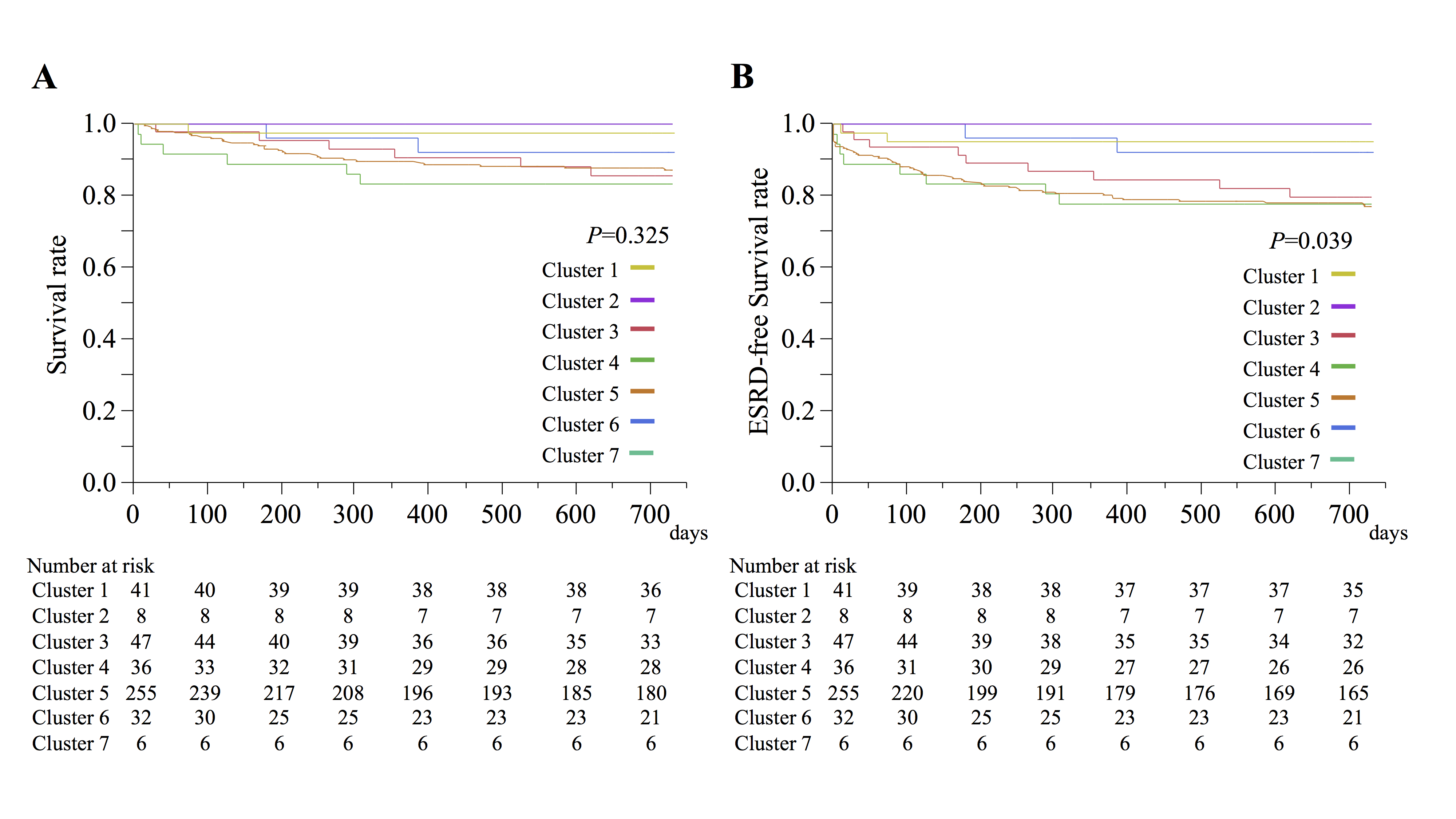

Supplement: Supplementary file 4 — Supplementary Figure S3. [file 41598_2021_84627_MOESM4_ESM.tiff]

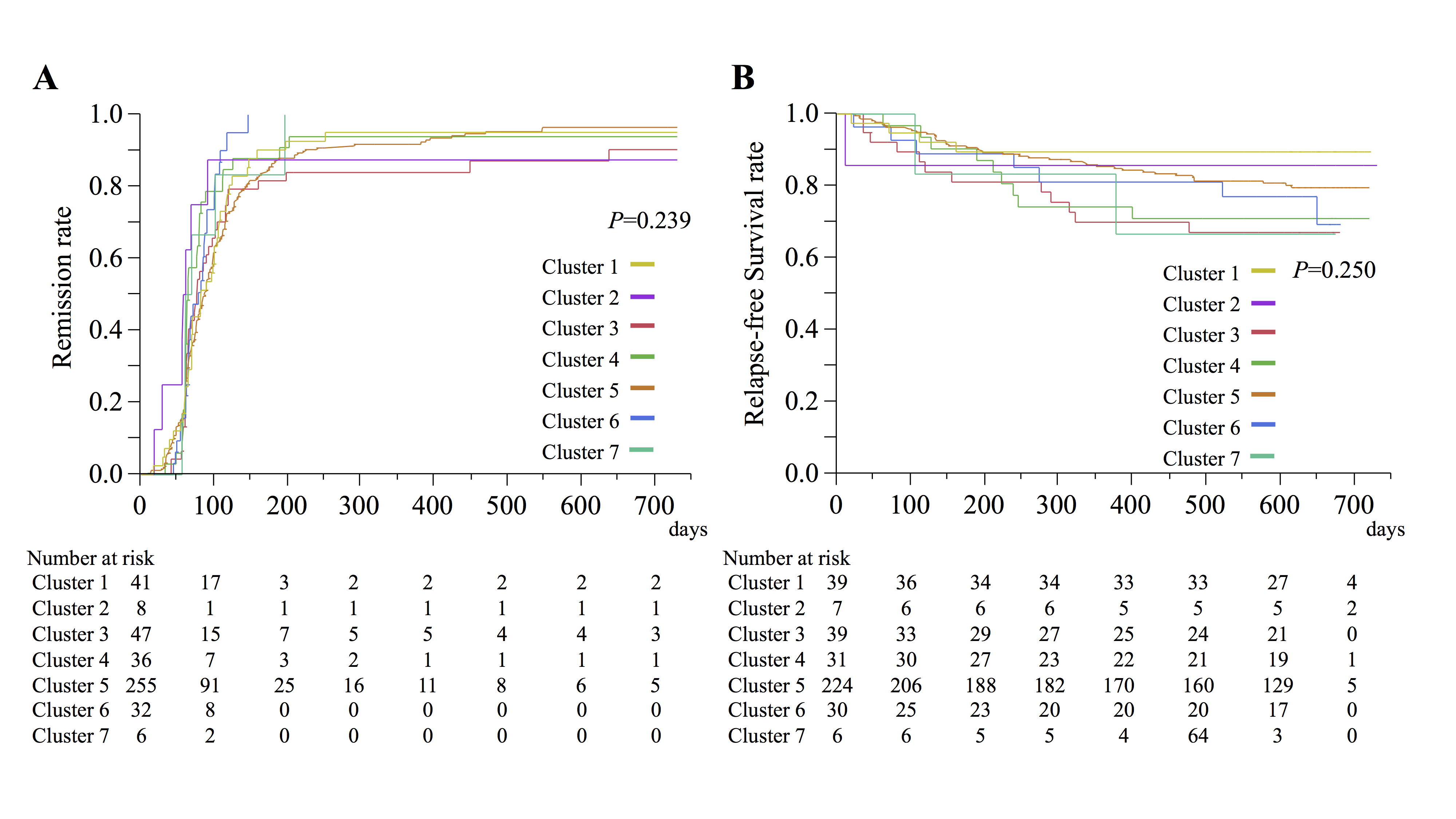

Supplement: Supplementary file 5 — Supplementary Figure S4. [file 41598_2021_84627_MOESM5_ESM.tiff]

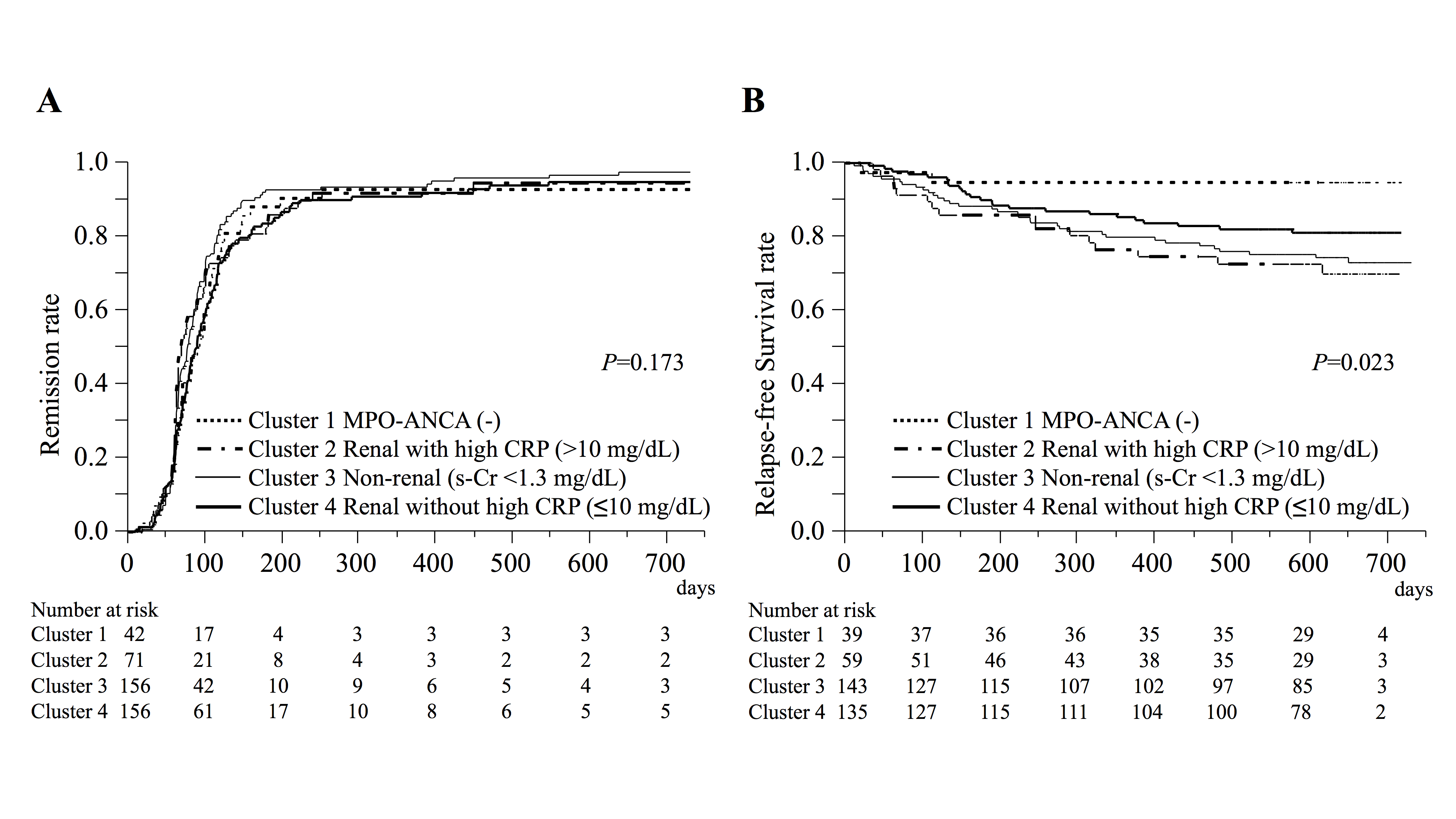

Supplement: Supplementary file 6 — Supplementary Figure S5. [file 41598_2021_84627_MOESM6_ESM.tiff]
